# Quantifying the Speed of Chromatophore Activity at the Single-Organ Level in Response to a Visual Startle Stimulus in Living, Intact Squid

**DOI:** 10.3389/fphys.2021.675252

**Published:** 2021-06-18

**Authors:** Stavros P. Hadjisolomou, Rita W. El-Haddad, Kamil Kloskowski, Alla Chavarga, Israel Abramov

**Affiliations:** ^1^Department of Social and Behavioral Sciences, College of Arts and Sciences, American University of Kuwait, Salmiya, Kuwait; ^2^Department of Psychology, Brooklyn College, City University of New York, Brooklyn, NY, United States

**Keywords:** cephalopod, chromatophore, camouflage, communication, body pattern, startle response, light flash stimulation, temporal dynamics

## Abstract

The speed of adaptive body patterning in coleoid cephalopods is unmatched in the natural world. While the literature frequently reports their remarkable ability to change coloration significantly faster than other species, there is limited research on the temporal dynamics of rapid chromatophore coordination underlying body patterning in living, intact animals. In this exploratory pilot study, we aimed to measure chromatophore activity in response to a light flash stimulus in seven squid, *Doryteuthis pealeii*. We video-recorded the head/arms, mantle, and fin when squid were presented with a light flash startle stimulus. Individual chromatophores were detected and tracked over time using image analysis. We assessed baseline and response chromatophore surface area parameters before and after flash stimulation, respectively. Using change-point analysis, we identified 4,065 chromatophores from 185 trials with significant surface area changes elicited by the flash stimulus. We defined the temporal dynamics of chromatophore activity to flash stimulation as the latency, duration, and magnitude of surface area changes (expansion or retraction) following the flash presentation. Post stimulation, the response’s mean latency was at 50 ms (± 16.67 ms), for expansion and retraction, across all body regions. The response duration ranged from 217 ms (fin, retraction) to 384 ms (heads/arms, expansion). While chromatophore expansions had a mean surface area increase of 155.06%, the retractions only caused a mean reduction of 40.46%. Collectively, the methods and results described contribute to our understanding of how cephalopods can employ thousands of chromatophore organs in milliseconds to achieve rapid, dynamic body patterning.

## Introduction

Unlike the slower chromatophore control of flatfish (2–8 s; Ramachandran et al., 1996), coleoid cephalopods can change body patterns in milliseconds. For decades, scientists in the field of cephalopod vision have focused on the goal of creating a complete characterization of the sophisticated coleoid body patterning abilities. As a result, existing reports are sufficient to describe and explain several known body patterns in cephalopods for camouflage and communication (Hanlon and Messenger, 1988; Hanlon, 2007; Langridge et al., 2007; Zylinski et al., 2009; How et al., 2017). Nevertheless, a theoretical framework on cephalopod body patterning, which does not include the dimension of time, will be inherently inadequate in modeling, holistically, the range of dynamic, rapid transformations observed in animals living in the wild. One approach toward studying this topic is by stimulating the visual system of a living, intact animal, using a light flash to elicit muscular activation of chromatophores, and quantifying the response dynamics by tracking surface area changes in time.

Experiments conducted in the Gilly laboratory revealed how light flashes elicit startle jet-escape responses in squid, *Doryteuthis opalescens* (Berry, 1911). The brief, intense light stimulus activates the central nervous system (CNS) at the magnocellular and palliovisceral lobes, which relay information to the stellate ganglia to modulate forceful muscle contractions of the mantle expelling water through the funnel in the process (Otis and Gilly, 1990; Gilly et al., 1991; Gilly and Lucero, 1992; Neumeister et al., 2000; Preuss and Gilly, 2000). Within the stellar nerve, a group of non-giant motor axons innervates chromatophore muscles (Ferguson et al., 1988). In one of these studies (Neumeister et al., 2000), which investigated the effects of temperature on escape responses in restrained squid, the flash stimulus produced transient chromatophore expansions. Responding to the light flash startle stimulus, animals exhibited a robust jet-escape startle response with transient chromatophore expansions. However, when light intensity was decreased by “positioning the flash unit further from the squid” (Neumeister et al., 2000, p. 551), the animal showed chromatophore expansions as *sub-jet-threshold* startle responses (in the absence of jetting). Squid are a useful species for studying chromatophores because they have fewer and larger chromatophore organs (density: 8 mm^–2^, maximum diameter: 120–1,520 μm; Hanlon, 1982) compared to octopus (density: 230 mm^–2^; maximum diameter: 300 μm; Packard and Sanders, 1971) and Sepia (density: 35–50 mm^–2^; maximum diameter: 300 μm; Hanlon and Messenger, 1988), offering a simpler model to study chromatophore control.

The Neumeister et al. (2000) study validates a reliable method of using flash stimulation and video-recording the skin, from a close-up perspective, to investigate the synchronicity of chromatophore activity at the single-organ level in squid. Since studying chromatophore response dynamics across all body regions was not the study’s primary focus, chromatophore expansions only on the mantle were reported. For this exploratory pilot study, we aim at replicating the sub-jet-threshold behavioral responses to flash stimulation with a different species, *Doryteuthis pealeii* (Lesueur, 1821), to examine the mechanisms and temporal dynamics of the sensorimotor system underlying chromatophore control in intact animals (Hadjisolomou, 2017). Due to ethical and logistical issues involved with long-distance transportation of *D. opalescens* for experimentation, *D. pealeii* was chosen as this species is available to be studied in Woods Hole, Massachusetts.

**FIGURE 1:**
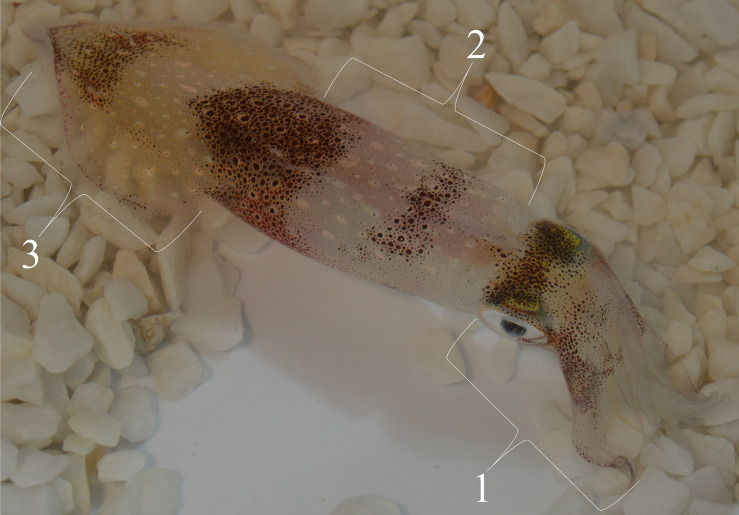
*D. pealeii* (mantle length approximately 14 cm) expressing disruptive body patterning with some chromatophores expanded (dark bands), while others are retracted. Numbers indicate the different body regions measured in the study: 1 = head/arms, 2 = mantle, and 3 = fin.

Further, in addition to the mantle, we expanded observations to include chromatophore activity from the understudied regions of the arms, head, and dorsal fin (Figure 1). Young (1976) reported on the CNS control of chromatophores in *D. pealeii*, elaborating that separate chromatophore lobes in the brain control different body regions. Specifically, the posterior chromatophore lobes (PCL) mainly control chromatophores on the mantle and fin regions, while chromatophores on the arms and head are primarily controlled by the anterior chromatophore lobes (ACL) and pedal lobes (PL). Axons from the PCL connect without a synapse to chromatophore organs through the pallial nerve. Electrode stimulation of PCL neurons in *Lolliguncula brevis* (Blainville, 1823) causes chromatophore expansion on the mantle and fin (Dubas et al., 1986), but it did not result in retraction of any expanded chromatophores. Both species are part of the same family, Loliginidae (Lesueur, 1821), and have anatomical similarities (Díaz-Santana-Iturrios et al., 2019), thus allowing for approximations between them. We chose these body regions to observe any discrepancies in timed responses due to circuitry differences. By video-recording all body regions in intact, living squid, we quantified the temporal dynamics from light flash stimulation to expansions and retractions at the single-organ level across thousands of chromatophores. Similar to the Reiter et al. (2018) study, which used unrestrained European cuttlefish (Linnaeus, 1758), we measured chromatophore activity from unrestrained squid. The procedures and methodologies described below enable non-invasive data collection of chromatophore activity from living animals to study behavioral responses in intact organisms.

## Methods

### Animals

Adult *D. pealeii* were collected from coastal waters near Woods Hole, Massachusetts, US, in 2014. From large population holding tanks, eight healthy animals (mantle length: 12–15 cm; unknown sex and age) without any visible physical injuries were selected for inclusion. We transported individual squid and housed them together in a 2 m × 1.5 m × 1 m rectangular, light-brown opaque, fiberglass housing chamber connected to an open, temperature-controlled (17–19°C) seawater system. Gravel and sand on the bottom of the housing tank provided a natural substrate for animals to settle. Animals were fed twice a day on an *ad libitum* diet of live *Fundulus* fish and crabs^1^.

### Experimental Design

Here, *n* refers to the number of different body regions examined (head/arms, mantle, and fin). Each body region, therefore, was considered to be an experimental unit. The study was a within-subjects design consisting of one group of three experimental units, and there were eight animals. One animal was excluded due to a lack of significant chromatophore responses from data analysis (see “Results” section).

### Procedure

#### Experimental Set-Up

To collect measurements, we constructed a rectangular rig covered with a layer of black cloth and an additional layer of an opaque, black tarp to prevent light from entering.

#### Experimental Tank and Acclimation

**FIGURE 2:**
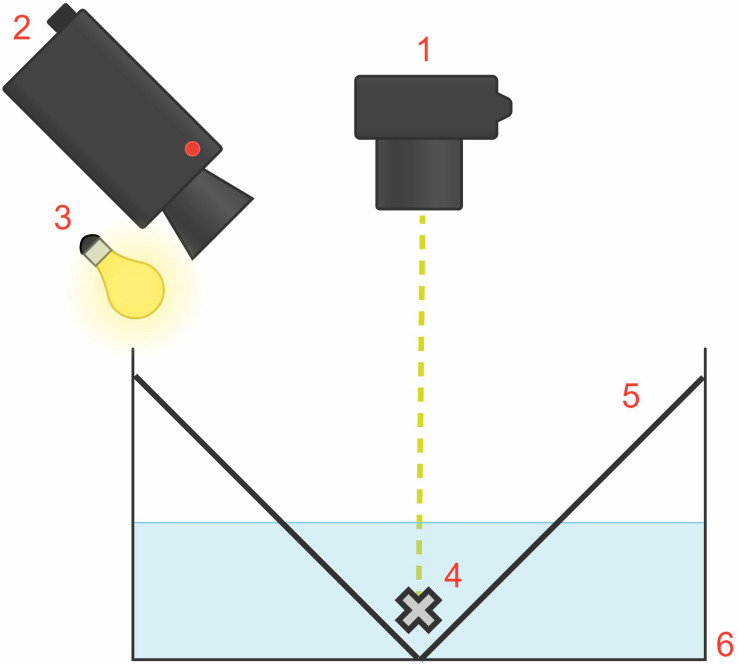
Diagram of the experimental tank set-up, measuring 53 cm × 43 cm × 18 cm (situated inside the rig; external rig structure and black tarp and opaque covers not shown). The flash unit (1) providing the visual startle stimulus was fixed on the rig at a right angle and 50 cm above the animal (4). The camera (2) and light source (3) were at a 45° angle above the animal. The white “V-shaped” partition configuration (5) enabled squid to settle naturally at the bottom of the white, rectangular tank (6).

The rectangular experimental tank, measuring 53 cm × 43 cm × 18 cm, consisted of white, opaque plastic walls containing 10 L of seawater (Figure 2). For each trial, one squid was placed within the experimental tank inside the rig. To establish habituation to the experimental apparatus, each squid was placed in the experimental tank for 10 min then returned to the group home tank, 24 h before experimental trials began. We created a white “V-shaped” partition configuration to enable the squid to settle naturally at the bottom of the tank, thus preventing chromatophore displacement outside of the camera frame. We placed an overhead light source at a 45° angle to illuminate the animal for video recording. The ambient light and visual environment determined the chromatophore’s state (expanded or retracted) before light flash stimulation. The animals adopted a lighter skin tone to camouflage in the white, uniformly lit tank during trials. Thus, to allow for a lighter skin tone, most chromatophores were retracted before flash stimulation.

#### Startle Stimulus and Sub-Jet-Threshold Startle Response

Animals were presented with light flashes to elicit the startle reflex response. To deliver the startle stimulus in a top-down direction, a Canon SpeedLite 580EX-RT flash unit was fixed on the rig at a right angle and 50 cm above the animal. Similar to the Neumeister et al. (2000) study, we found that *D. pealeii* have jet-escape startle responses and transient chromatophore expansions to intense light flashes. For this study, the duration of each light flash stimulus was ∼100 μs, with an illuminance of 12,500 lx, providing an even exposure of the stimulus on the animal from this distance. The entire animal was illuminated, but we video-recorded only one specific body region per trial for analysis. The stimulus was sufficient in producing muscular contraction but well below the jet-escape sensory threshold to minimize jetting. Thus, this study’s behavioral responses comprised of chromatophore expansions and retractions to light flashes in the absence of jetting.

#### Experimental Trial Procedure

Once in the experimental rig, animals were allowed to procedurally acclimate and settle on the bottom of the tank, as evidenced by the animal remaining motionless for at least 5 min. Once an animal habituated, it received a sequence of approximately 90 flashes. For this study, we used a 10-s inter-stimulus interval (ISI), which does not cause attenuation due to learning, fatigue, or a combination of both (Otis and Gilly, 1990). With an ISI of 10 s, the total sequence duration lasted for 15 min per body region and each region was tested during different sessions. The duration and ISI were tested in preliminary trials and found to be appropriate for the purposes of this study. The rationale was to reduce testing sessions and have only one per body region since 15 min were sufficient.

Each flash stimulation was considered an individual trial. The purpose was to elicit the sub-jet-threshold startle response. Each animal received 90 trials for each of the three body regions, for a total of 270 trials for each of the eight animals, thus 2,160 trials in total. One body region per animal was tested at a time (we counterbalanced the order of the body regions tested per animal).

For details on video-recording, scoring, image analysis, and statistical analysis see Supplementary Material.

## Results

### Chromatophore Surface Area Changes Following Light Flash Presentation

Out of the 2,160 total trials, 230 were suitable to be analyzed by Change Point Analysis (CPA) (Taylor, 2000). Based on CPA, 185 were identified to have significant chromatophore surface area changes. A total of 4,065 individual chromatophores responded to the startle stimulus with either transient expansion or retraction of the pigment. These chromatophores were further analyzed to characterize response activity pre- and post-stimulation. The remaining 45 trials showed no significant responses by CPA and were excluded, including all Squid #8 trials and all expansion trials in Squid #3.

Additionally, the numbers of trials with chromatophore responses were not equivalent across squid (Squid #2, for example, did not show any retraction responses in any trials). Furthermore, not all squid had all body regions significantly responding to the flash stimulus, and in other cases, there were trials with both significant expansion and retraction instances on the same body region. Thus, there is an unequal distribution of chromatophore numbers and body regions represented in the data (see Supplementary Figures 2–5).

The discrepancies in this dataset are the observed behavioral differences between animals; a few animals would swim back and forth often enough to invalidate significant parts of the footage. Additionally, trials were excluded in the process of image analysis if the software was unable to detect chromatophores (Hadjisolomou and El-Haddad, 2017). In such cases, image noise due to fluctuations of color and luminance created artifacts that interfered with chromatophore detection and tracking. However, each significant expansion or retraction followed the same pattern regardless of which body region or squid showed the response.

### Chromatophore Expansion

Out of the 185 trials with significant chromatophore surface area changes (from seven animals), 166 (thus, 90% of these trials) showed expansion following the flash stimulus (six animals). Out of these, the head/arms region had 85 trials (Squids #1, 5, and 7), followed by the mantle with 50 (Squids #1 and 4–7), and then the fin with 31 (Squids #2 and 5–6).

Within these 166 trials, 4,000 (98% out of the total 4,065) chromatophores showed significant expansion. On the head/arms, there were 1,598 chromatophores; from the mantle, there were 1,743; and on the fin, there were 659.

### Chromatophore Retraction

The remaining 19 trials showed significant chromatophore retractions following the flash stimulus (six animals). The head/arms had nine trials (Squids #1, 3, and 7), the mantle eight (Squids #1 and 4–6), and the fin had two (Squid #1).

Within these 19 trials, we tracked and measured 65 chromatophores showcasing significant retraction. On the head/arms, there were 39 chromatophores; from the mantle 21; and on the fin, there were five.

### Descriptive Statistics of Transient Responses

#### Temporal Dynamics

**FIGURE 3:**
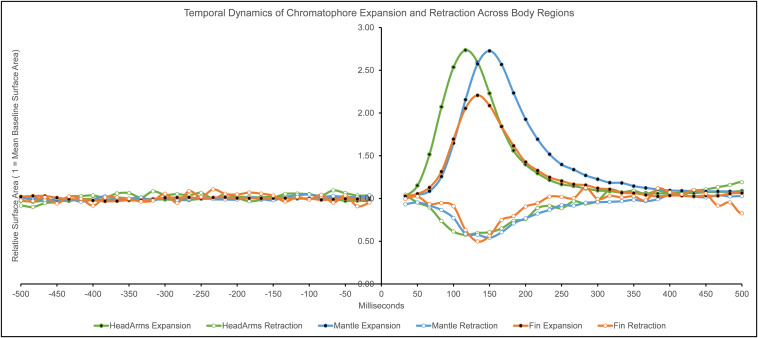
Temporal dynamics (in milliseconds) of chromatophore expansion and retraction across body regions. Surface area values are relative to the average pre-flash chromatophore surface area (1 = average of pre-flash chromatophore surface area; values above 1 = expansion, values below 1 = retraction). Negative values of milliseconds indicate time before the light flash presentation, 0 indicates flash presentation, positive values indicate time after the light flash presentation (The two frames containing the flash stimulus, *t* = 0 ms and t = 17 ms, were removed from analysis due to the animal not being observable).

We calculated descriptive statistics on the temporal dynamics of chromatophore surface area changes following the startle stimulus (see Table 1 and Figure 3). We estimated each value with an estimated margin of error of ± 16.67 ms, determined by the inter-frame interval when recording at 60 frames per second frequency.

**TABLE 1:** Descriptive statistics of temporal dynamics in milliseconds (ms).

	Response time (tR)		Delay time (tD)		Rise time (tRt)		Response duration (rD)	
	Expansion (ms)	Retraction (ms)	Expansion (ms)	Retraction (ms)	Expansion (ms)	Retraction (ms)	Expansion (ms)	Retraction (ms)
Head/Arms	50	50	83	83	117	117	300	267
Mantle	67	67	117	100	150	150	384	250
Fin	67	67	100	117	134	134	334	217

*Response time (tR) is the time to reach or pass the 5% value of the maximal response;delay time (tD) is the time to reach or pass the 50% value of the response; rise time (tRt) is the time required to reach the 100% value of the response; response duration (rD) is the time between the 5% values of response before and after the peak.*

#### Magnitude of Response

We calculated the magnitude of chromatophore expansion or retraction activity by comparing peak response values of response chromatophore surface area (RCSA) with the pre-flash baseline chromatophore surface area (BCSA) (Figure 3).

##### Expansion

On average, the relative chromatophore surface area increased by 155.06% across all body regions (4,000 chromatophores). Specifically:

Head/arms: 159%

Mantle: 168%

Fin: 116%

##### Retraction

On average, the relative chromatophore surface area decreased by 40.46% across all body regions (65 chromatophores). Specifically:

Head/arms: 43%

Mantle: 46%

Fin: 50%

## Discussion

In this exploratory pilot study, we systematically elicited behavioral responses using a light flash stimulus in intact, living squid and analyzed the temporal dynamics and magnitude of thousands of chromatophore surface area changes at the single-organ level. Here, we report a replication of the following Neumeister et al. (2000) findings using a different squid species. *D. pealeii* with uniform light skin patterns before stimulation responded to flashes with jetting and chromatophore expansion and lower flash intensities triggered transient darkening in the absence of jetting. Our results demonstrate that it is feasible to use intact, living animals, to measure, non-invasively, the temporal dynamics of chromatophore control during body patterning.

We also report the following novel observations: this is the first record of chromatophore activation to light flash stimulation on regions other than the dorsal mantle; our videos show chromatophore activation on the head, arms, and fin, in addition to the mantle. Also, for the first time, we show chromatophore retraction to light flash stimulation: chromatophores that were expanded before stimulation (such as dark bands on the mantle or expanded chromatophores on the head) responded with a transient retraction. Further, we observed synchronous chromatophore expansion and retraction on different parts of the mantle in the same trial (for example, chromatophores on dark bands on the mantle contracted, while chromatophores on light skin expanded).

The general temporal dynamic patterns emerging from this data are the following: the speed of expansion and retraction activation was the same across body regions. Differences in response durations were not dependent on the magnitude of response. Finally, the head/arms were faster in most measurements compared to other body regions. The short latencies reported here are suggestive of a reflexive component of the response.

### Light Flash Stimulation Elicits Sub-Jet-Threshold Responses in *D. pealeii*

#### Chromatophore Expansion

Packard and others have described how flashes of light can elicit responses in chromatophores from dissected octopus skin (Packard and Brancato, 1993; Ramirez and Oakley, 2015). More relevant to this study, Neumeister et al. (2000) reported how light flashes elicit sub-jet-threshold chromatophore expansion in the squid *D. opalescens*. In agreement with the Neumeister study findings, we demonstrate that the presentation of light flashes elicits chromatophore expansion in a different squid species, *D. pealeii*. Also, we validate a method to measure chromatophore activity from unrestrained squid.

#### Chromatophore Retraction

This is the first study to report chromatophore retraction in response to presentation of a light flash stimulus. Comparing the two different types of chromatophore activity, expansions and retractions, enables a more thorough characterization of the sensorimotor system since the mechanisms underlying each type of action are not well understood. However, out of 4,065 chromatophores analyzed, only 65 showed retraction. As stated in the “Experimental Tank and Acclimation” section, only a small number of chromatophores were expanded in the original experimental set-up. Therefore, chromatophores responded in the only possible outcome given their original state: retracted chromatophores expanded and expanded chromatophores retracted. These findings demonstrate the method’s validity in studying the retraction mechanism, an essential part of the chromatophore system in rapid body patterning.

### Characterization of Temporal Dynamics of Sub-Jet-Threshold Responses

#### Response Time (tR)

Our findings indicate similarities when comparing expansion with retraction and between the different body regions. The average response time to reach or pass the 5% value of the maximal response was 50 ms (± 16.67 ms). This was identical across all body regions and between expansions and retractions. These results echo the timing of the startle response mentioned in previous studies (Neumeister et al., 2000; Mooney et al., 2016). Based on these findings, the speed of the onset of rapid body patterning in squid is characterized by a latency of 50 ms.

#### Delay Time (tD)

When measuring the average time to reach or pass the 50% value of the response, the head/arms is faster in reaching this mark than the other two body regions in both expansions and retractions. We believe this difference can be explained by the fact that chromatophores on this body region are controlled by separate lobes (ACL and PL; Young, 1976), and thus the temporal discrepancies may be due to the circuitry. The differences between fin and mantle timings average out when we aggregate data for both expansion and retraction.

#### Rise Time (tRt)

The rise time to reach the 100% value of the response peak is the same within body regions in expansion and retraction, though there are differences between regions. Thus, each body region has specific temporal benchmarks of maximum response regardless of the chromatophore change type. The chromatophores on the head/arms are the fastest between body regions, followed by the fin in second place, and lastly, the mantle. Considering the slight differences in the magnitude of response between the body regions, it is surprising that the chromatophores on the head/arms are about 33 ms faster than those on the mantle. The time difference may not be explained due to response magnitude since these two body regions are almost identical in that dimension. The circuitry’s differences (Young, 1976) may explain these temporal discrepancies on the head/arms (ACL and PL) compared to those of the mantle (PCL).

#### Response Duration (rD)

Most discrepancies were found in the response duration, the time between the 5% values of response before and after the peak, between and within body regions when comparing expansion and retraction. We calculated the duration by finding the time difference between the initial response and the return to the pre-flash state following the peak response. Across the type of responses and body regions, chromatophore change duration is short, between 217 and 384 ms. Compared to color changes seen in other species (Ramachandran et al., 1996), the sub-second cephalopod chromatophore change is unparalleled.

When it comes to expansion, the chromatophores on the head/arms are the fastest to complete the response and reach pre-flash surface area values at 300 ms, followed by the fin (+34 ms) and the mantle (+84 ms). A different pattern was observed with retraction responses: chromatophores on the fin had the shortest duration of response at 217 ms, followed by those on the mantle (+33 ms), and lastly by those on head/arms (+50 ms).

It is worth noting that the response duration was the only dimension in which retraction had a shorter overall interval than expansion. For example, the most prolonged response duration during retraction (267 ms) was still faster than the briefest response duration in expansion (300 ms). One reason to explain this phenomenon is that chromatophore expansion and retraction may depend on separate mechanisms; during expansion, the surrounding radial muscles pull and expand the pigment (Bell et al., 2013). The retraction mechanism, however, is still not fully understood.

### Characterization of the Magnitude of Sub-Jet-Threshold Responses

Results indicate differences between the scale of chromatophore surface area changes when a chromatophore expands or retracts. While the surface area increased 155.06% on average during an expansion, the retraction only caused a 40.46% decrease. As discussed in the “Response Duration” section, one reason for this may be the different mechanisms involved in expansion compared to retraction.

Other discrepancies were found when analyzing chromatophores across the body regions. The mantle and head/arms showed the largest surface area expansions with 168% and 159% corresponding changes, respectively. The fin had a 116% increase on average. It is unclear why there is a close to 50% difference between the fin and the other regions. This may be due to differences in the type and distribution of chromatophores on the fin compared to the head/arms and mantle when it comes to body patterning. It is necessary to investigate further if fin chromatophores do not expand as much as those on the mantle and head/arms and why that would be the case.

## Limitations and Future Directions

### Unequal Distribution of Trials Between Body Regions and Animals

The number of trials with significant chromatophore responses was not equal per body region within each animal nor between animals, and thus there was an unequal distribution of body regions and chromatophores represented in the dataset. This unequal distribution precludes the possibility to run statistical analyses in determining significant differences in the temporal dynamics and magnitude of responses. Also, due to ethical considerations, we determined that a larger number of animals to be used was not well-warranted. For future studies, we advise scheduling shorter trials over several days so more data can be collected from fewer animals.

### Unequal Number of Significant Surface Area Changes Between Expansion and Retraction

Out of the 4,065 chromatophores showing significant responses, only 65 showed retractions. The small sample size makes it difficult to generalize the retraction results. To promote the animal adopting a darker skin tone, we ran additional pilot trials using black tanks and white gravel to generate visual contrast between the substrate and walls. The contrast increased the probability of squid expressing a disruptive or uniformly dark pattern. When squid experienced light flashes while having dark patches of skin, we observed more retractions. However, attempting to replicate these trials using black tanks *within* the rig was impossible due to the video frames’ noise resulting from less visibility. Future studies on chromatophore retraction may utilize visual contrast in the environment and appropriate equipment to remove videography noise.

### Potential Extraocular Chromatophore Responses

The overall results of our study showed that the response time (tR) was in line with timings from Otis and Gilly (1990). They argue that “[t]he 50-ms delay for giant axon excitation in the startle-escape is similar to that for mantle contraction, indicating that the major source of behavioral delay lies in the central nervous system and not in conduction time along the giant axon (<10 ms) or muscle activation” (p. 2912). Thus, we may conclude that squid chromatophore responses are dependent on the CNS. To test the possibility that squid skin responds directly to light flashes, we used flash stimulation with a recently deceased squid from the main population holding tank. The squid showed spontaneous chromatophore activity before stimulation, and the aim was to observe if there were any extraocular chromatophore responses to the flash stimulus. We found no discernible changes due to stimulation. However, since we only used one deceased squid to test this, we cannot exclude the possibility that extraocular responses may have contributed to chromatophore activity changes in this study.

## Conclusion

In the natural world, cephalopods are renowned for the dynamic range and speed of adaptive body patterning used in camouflage and communication. In this exploratory study, we used a light flash stimulus to elicit transient chromatophore surface area changes to quantify the chromatophore system’s temporal dynamics in living, intact animals. Our measurements here verify the early onset of the sub-second chromatophore changes in body patterning with an unparalleled speed. Based on our findings, we argue that measuring the temporal dynamics of complete behavioral responses during body patterning in intact, living animals is a feasible and essential addition to studies using excised isolated skin of subjects. The unexpected differences between body regions and expansion and retraction responses exemplify the need to continue this research line. Such details of timing the temporal dynamics are essential for comprehensive and quantitative descriptions of body patterning. The methodology and findings described in this study collectively contribute to our understanding of how cephalopods can employ thousands of chromatophore organs within milliseconds for rapid, adaptive body patterning.

## Data Availability Statement

The raw data supporting the conclusions of this article will be made available by the authors, without undue reservation.

## Ethics Statement

Ethical review and approval was not required for the animal study because Ethical approval was not required since, at the time this study took place (July, 2014), the Institutional Animal Care and Use Committee (IACUC) protocols were not issued for invertebrate research in the United States and in the Institution where the experiments with live animals were carried out. Nevertheless, procedures were performed to minimize pain and distress of the animals involved.

## Author Contributions

SH, KK, AC, and IA contributed to the conception and design of the study. SH ran the video trials, collected data, organized the database, and wrote the manuscript’s first draft. SH and RE-H performed the statistical analysis. RE-H wrote sections of the manuscript. All authors contributed to manuscript revision, read, and approved the submitted version.

## Conflict of Interest

The authors declare that the research was conducted in the absence of any commercial or financial relationships that could be construed as a potential conflict of interest.

## Footnotes

1 See Supplementary Material for the Ethical Note and Experimental Controls sections.
