# Supplementary material for: Quantifying the Speed of Chromatophore Activity at the Single-Organ Level in Response to a Visual Startle Stimulus in Living, Intact Squid
Source: Front Physiol. 2021 Jun 18;12:675252. doi: 10.3389/fphys.2021.675252 (PMC8250766; doi:10.3389/fphys.2021.675252)
Supplement: Supplementary file 5 [file Data_Sheet_1.docx]

Supplementary Material

# Ethical Note

Ethical approval for this study was not required since, at the time this study took place (July 2014), the Institutional Animal Care and Use Committee (IACUC) protocols were not issued for invertebrate research in the US and in the institution where the experiments with live animals were carried out. Nevertheless, the following procedures were performed to minimize the pain and distress of the animals involved.

For ethical reasons, we used a minimal number of animals to collect a sufficient amount of data to establish appropriate and sufficient procedure and equipment parameters in achieving novel behavioral measurements. Animals were acclimated for 48 hours before being habituated to the experimental apparatus. One day following habituation, experimental trials began. Gravel and sand on the bottom of the housing tank provided a natural substrate for animals to settle. To minimize distress, we did not measure the animals’ weight. A 12/12 h light/dark cycle was maintained, and the housing tank was cleaned twice a day. Each animal was gently transported from the housing tank to the experimental rig using a glass beaker (to minimize distress, we did not use nets), with this task lasting approximately one minute. At the end of each testing session, the animal was returned to the housing system. Animals were kept in the housing system for the study duration (11 days) and remained there after study completion.

Squid were monitored twice daily by caretaking staff and the first author throughout the study duration. Animal feeding and group interaction behavior were observed, as well as if they had developed any injuries. Had an animal shown changes in feeding or group interaction, trials would have been terminated for the animal, and it would have been excluded from data analysis. Had an animal developed any physical injuries, it would have been euthanized. However, no adverse events were observed or recorded. All animals completed the study without any injuries or changes in feeding or group behavior. All animals remained in their housing tank after the completion of the study.

# Experimental Controls

Since this pilot study intended to test the feasibility of a procedure, there was no single primary outcome (multiple outcomes were assessed) and did not require a control group or randomization (Percie du Sert et al., 2020).

Since all animals received the same stimulus, no blinding process was necessary as the light flash stimulus illuminated the whole animal body (all three body regions) in all trials. All conditions (housing, transportation to experimental tank, experimentation) were the same for all animals. The animal caretaking staff and first author were involved with all animal care tasks, and only the first author was involved in experimental procedures.

Experimental measurements began each day at noon, and the experimenter was not aware of the behavioral results until scoring and analysis were performed at a later stage since the animal was concealed in the rig during data collection. The flash unit was triggered remotely using a wireless device so that the animals would not experience visual or auditory contact with the experimenter. Furthermore, the chromatophore surface area changes were measured automatically using the *SpotMetrics* software plugin, which provided unbiased data scoring (Hadjisolomou & El-Haddad, 2017). Further, change-point analysis (see Change-point analysis section) software analyzed the *SpotMetrics* output data and provided automated unbiased assessments of significant chromatophore surface area changes.

# Data Acquisition and Statistical Analyses

## Video-recording. We used one Nikon D5300 camera on a tripod pointed at the animal (45°) to video-record behavioral responses at 60 frames per second (fps) and a 1280 × 720 pixels resolution (720p) to capture individual chromatophore activity before and after the presentation of light flashes. We recorded multiple videos of each body region separately: head/arms, mantle, and fin.

Video selection. We reviewed all trials to exclude the ones not suitable for analysis due to excessive animal movement. In such cases, chromatophores would not be trackable throughout the trial since they would move in and out of the reference frame and produce incomplete datasets. Out of the 2,160 trials, there were 716 deemed suitable for scoring to detect sub-jet-threshold startle responses (see Supplementary Table 1).

Video scoring. Of the 716 trials, 522 were determined to be suitable for image analysis. Inclusion criteria required that videos showed a visible chromatophore response in addition to the animal not moving significantly before or after the flash stimulus (see Supplementary Table 2).

Image analysis. We edited, compiled, and rendered sequences of frames showcasing multiple individual chromatophores in the *Audio Video Interleave* (AVI) format using the video analysis suite *Sony Vegas Pro 13*. The AVI format was chosen specifically for the *Fiji Is Just ImageJ* (FIJI) image analysis software used in chromatophore detection and tracking. Each frame contained a 2 cm × 3 cm (6 cm^2^) area of skin. Each video contained frames capturing one second before the flash, the flash instance, and one second after the flash. Since we recorded chromatophore videos at 60 fps, each video contained 121 frames with an interval of 16.67 milliseconds.

Chromatophore surface area analysis. The following parameters were assessed: baseline chromatophore surface area (BCSA) as one second of videography (60 continuous frames) of surface area measurement in pixels before flash stimulation; response chromatophore surface area (RCSA) as one second of videography (60 continuous frames) of surface area measurement in pixels of chromatophore surface area after the flash simulation. We used a FIJI image analysis software plugin, *SpotMetrics*. *SpotMetrics* processed thousands of frames from high definition (HD) videos; it automated detecting, tracking, and analyzing thousands of individual chromatophores over time (Hadjisolomou & El-Haddad, 2017). On average, there were 1,000 visible chromatophores in one frame. Once the plugin analyzed all individual chromatophore surface area changes, it automatically exported data as an Excel spreadsheet. We used *SpotMetrics* to analyze every trial, generating Excel spreadsheets with individual time-series data from thousands of chromatophores. Of the 522 trials analyzed by *SpotMetrics,* a total of 230 were deemed suitable for change-point analysis based on at least one chromatophore being detected and trackable by the software (see Supplementary Table 3).

Change-point analysis. The *Change-point Analysis* (CPA) software (Taylor, 2000) was used to determine significant chromatophore surface area changes resulting from light flash stimulation (See Supplementary Figure 1). CPA analyzes a time series in search of any significant changes that cannot be explained by normal variability, with a minimum confidence level of 90%. It also detects the time step the changes take place with a 95% confidence interval. The CPA analysis software automatically checks the data to see if assumptions were met for the statistical approach.

Further, normal probability plots were used to visually evaluate data for normality. No data transformation was used since all the data met assumptions. We expected significant changes in the surface area of individual chromatophores following light flash stimulation. We analyzed every chromatophore using CPA software and collected all responsive chromatophores with significant surface area changes for further statistical analysis (See Supplementary Figures 2-5 for details of significant chromatophore expansion and retraction trials per animal and body region).

**Temporal dynamics of responses.** Since “speed” can refer to different temporal dynamics of the response analyzed here, we specify each assessed parameter with the following labeling. We calculated the response time (tR), the latency of the earliest noticeable increase (expansion) or decrease (retraction) of the chromatophore surface area, as the 5% mark of the maximal response (100%) change; delay time (tD), as the time to reach or pass the 50% value of the response; rise time (tRt), as the duration to reach the maximum response (100% of change); response duration (rD), the duration to return to baseline activity following earliest noticeable difference, was determined by the time between 5% values of response before and after the peak. We analyzed each trial consisting of significant chromatophore expansions or retractions resulting from flash stimulation to quantify the temporal dynamics of the response and magnitude of responses in Excel.

# Supplementary Figures and Tables

**Supplementary Table 1.** Number of trials (out of 90 per body region) deemed suitable for video scoring; videos with excessive movement were excluded.

|  | Head/Arms | Mantle | Fin | Total |
| --- | --- | --- | --- | --- |
| Squid 1 | 76 | 18 | 55 | 149 |
| Squid 2 | 46 | 4 | 34 | 84 |
| Squid 3 | 37 | 2 | 3 | 42 |
| Squid 4 | 13 | 24 | 12 | 49 |
| Squid 5 | 88 | 8 | 16 | 112 |
| Squid 6 | 21 | 75 | 24 | 120 |
| Squid 7 | 61 | 9 | 0 | 70 |
| Squid 8 | 68 | 17 | 5 | 90 |
| Total | 410 | 157 | 149 | 716 |

**Supplementary Table 2.** Number of trials showing a visible chromatophore response. These trials were entered into automatic image analysis, *SpotMetrics*.

|  | Head/Arms | Mantle | Fin | Total |
| --- | --- | --- | --- | --- |
| Squid 1 | 60 | 11 | 5 | 76 |
| Squid 2 | 41 | 3 | 23 | 67 |
| Squid 3 | 31 | 0 | 1 | 32 |
| Squid 4 | 7 | 9 | 8 | 24 |
| Squid 5 | 79 | 5 | 10 | 94 |
| Squid 6 | 10 | 67 | 15 | 92 |
| Squid 7 | 52 | 9 | 0 | 61 |
| Squid 8 | 54 | 17 | 5 | 76 |
| Total | 334 | 121 | 67 | 522 |

**Supplementary Table 3.** Trials with at least one chromatophore detected and trackable by *SpotMetrics* software. These trials were deemed suitable for change-point analysis.

|  | Head/Arms | Mantle | Fin | Total |
| --- | --- | --- | --- | --- |
| Squid 1 | 51 | 9 | 2 | 62 |
| Squid 2 | 0 | 0 | 17 | 17 |
| Squid 3 | 15 | 0 | 1 | 16 |
| Squid 4 | 1 | 4 | 4 | 9 |
| Squid 5 | 15 | 5 | 10 | 30 |
| Squid 6 | 5 | 25 | 7 | 37 |
| Squid 7 | 45 | 9 | 0 | 54 |
| Squid 8 | 0 | 0 | 5 | 5 |
| Total | 132 | 52 | 46 | 230 |


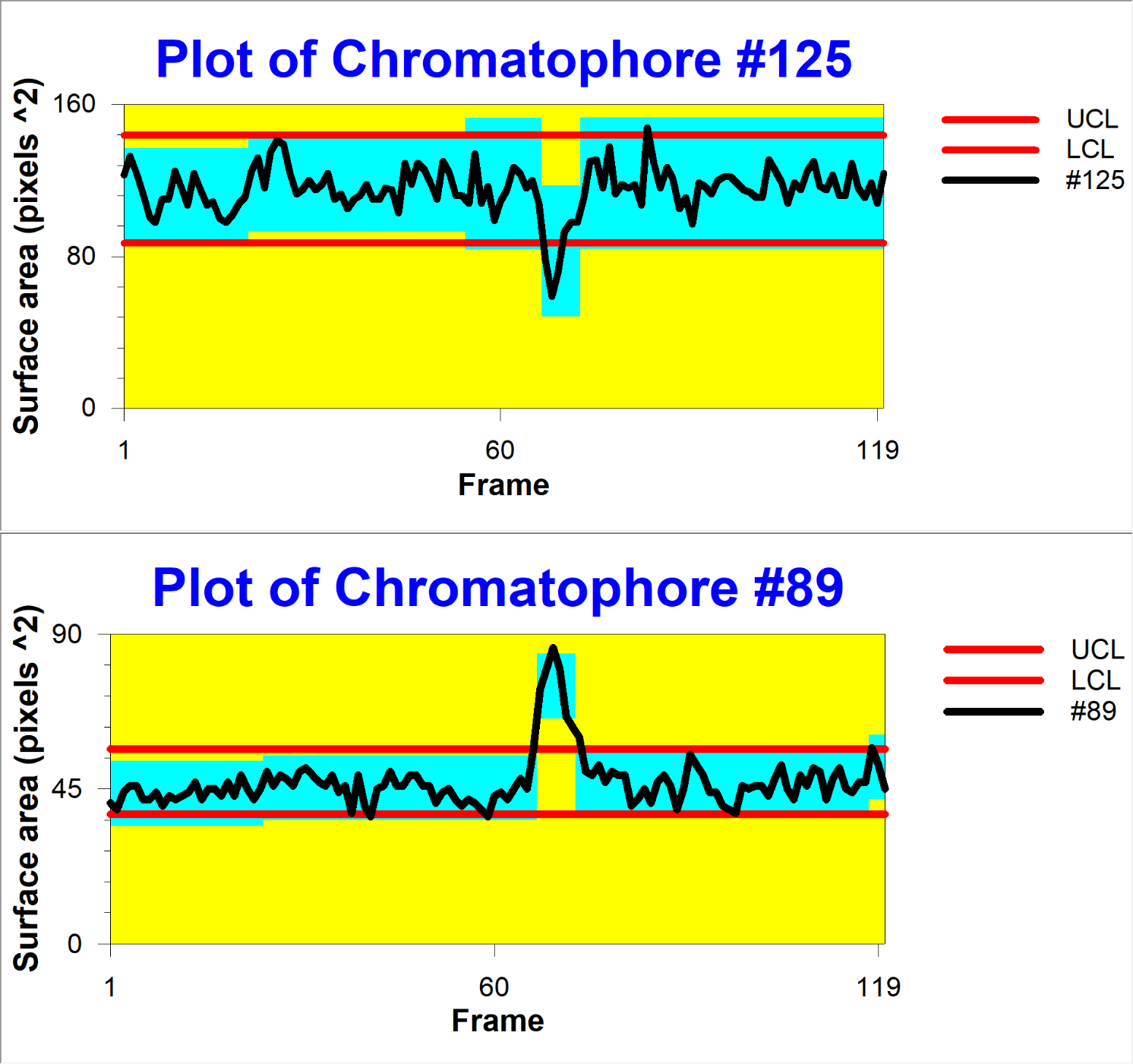


**Supplementary Figure 1.** Change-point analysis of retraction and expansion responses of two different chromatophores on the fin of the same animal. UCL = upper confidence level, LCL = lower confidence level (with a 90% confidence level).

**Supplementary Figure 2.** Trials showing significant chromatophore expansion responses per body region.

**Supplementary Figure 3.** Number of significantly expanded chromatophores per body region.

**Supplementary Figure 4.** Trials with significant chromatophore retraction responses per body region

**Supplementary Figure 5.** Number of significantly retracted chromatophores per body region

**References**

Hadjisolomou S. P. & El-Haddad G. (2017). SpotMetrics: An Open-Source Image-Analysis Software Plugin for Automatic Chromatophore Detection and Measurement. *Frontiers in Physiology, 8*(106).

Percie du Sert, N. P., Ahluwalia, A., Alam, S., Avey, M. T., Baker, M., Browne, W. J., ... & Würbel, H. (2020). Reporting animal research: Explanation and elaboration for the ARRIVE guidelines 2.0. *PLoS Biology*, *18*(7), e3000411.

Taylor, W. A. (2000). Change-point analysis: a powerful new tool for detecting changes. *Baxter Healthcare Corporation*. <http://www.variation.com/cpa/tech/changepoint.html>.
